# Comparison of intermittent and continuous proton pump inhibitor infusions in patients with non-variceal upper gastrointestinal bleeding at King Abdulaziz University Hospital, Jeddah, Saudi Arabia: A retrospective study

**DOI:** 10.15537/smj.2022.43.8.20220128

**Published:** 2022-08

**Authors:** Abeer S. Alzubaidi, Ahmed F. Basilim

**Affiliations:** *From the Pharmacy Department (Alzubaidi), and from the Department of Pharmacy Practice (Basilim), Faculty of Pharmacy, King Abdulaziz University, Jeddah, Kingdom of Saudi Arabia*.

**Keywords:** proton pump inhibitor, nonvariceal upper gastrointestinal bleeding, rebleeding, mortality, length of hospital stay

## Abstract

**Objectives:**

To compare the effectiveness of intermittent and continuous proton pump inhibitors (PPIs) infusion on the outcomes of patients with nonvariceal upper gastrointestinal bleeding (NVUGIB).

**Methods:**

The study was a single-centred retrospective study in adult patients with active upper gastrointestinal bleeding who received intermittent or continuous PPI infusion at King Abdulaziz University Hospital, Jeddah, Saudi Arabia, from January 2013 to October 2019. The outcomes assessed were rebleeding, length of hospital stays and mortality within 30 days of admission, and were compared between the treatment groups. A statistically significant *p*-value was set at 0.05.

**Results:**

The study involved 97 patients with NVUGIB treated with intermittent (n=56) and continuous (n=41) PPI infusions, with mean (±SD) ages of 66.0±16.1 and 58.0±19.5 years, respectively. The baseline and clinical characteristics between the 2 treatment groups; age (*p*=0.116), gender (*p*=0.345) and comorbidities (*p*=0.401), were comparable. There were no significant differences in rebleeding rates within 30 days (5 [8.9%] versus 1 [2.4%], *p*=0.396), length of hospital stays (4 vs 5, *p*=0.067), and mortality rate (1 [1.7%] vs 3 [7.3%], *p*=0.308) between the 2 groups.

**Conclusion:**

The management of NVUGIB with intermittent and continuous PPI infusions demonstrated comparable outcomes in reducing rebleeding rate, length of hospital stays, and mortality rate among patients attending the university hospital in Saudi Arabia.

**N**onvariceal upper gastrointestinal bleeding (NVUGIB) refers to the bleeding in the esophagus, stomach, and duodenum with hematemesis or melena presentation.^1^ The incidence of NVUGIB is between 50-150 per 100,000 adults per year, with a 2.5-10% mortality rate. The most prevalent causes of NVUGIB are peptic ulcers (28-59%), erosive mucosal disease (1-47%), Mallory-Weiss syndrome (4-7%), and upper gastrointestinal tract (GIT) malignancy (2-4%).^2^ The first step in managing NVUGIB is assessing the hemodynamic status and initiating resuscitative therapy.^3^ Endoscopic therapy is considered for patients with ulcer bleeding. For patients at high risk of bleeding ulcers, an initial intravenous (IV) bolus dose followed by continuous proton pump inhibitors (PPI) infusion therapy is recommended after endoscopic therapy.^3,4^ It is unclear whether substituting intermittent therapy for bolus plus continuous IV infusion PPI therapy would improve patient outcomes if it is as effective. Given the reduction in cost and resources, intermittent PPIs would be the preferred regimen if both achieve comparable efficacy. The most effective acid suppression should theoretically come from a high-dose continuous infusion. The most effective dosing and route of administration for PPIs in managing NVUGIB are still contentious.^5^

A previous study suggests that an intragastric potential of hydrogen (pH) of >6 improves clot formation and stability, and prevents recurrent ulcer bleeding.^6^ The targeted pH of >6 can be achieved with either an intermittent or continuous infusion of PPIs.^7^ In a trial of patients receiving endoscopic hemostasis for gastrointestinal (GI) bleeding, there was no significant difference between high and low-dose PPI infusion therapy regarding rebleeding, length of hospitalization, need for surgery, or mortality.^8^ Studies comparing intermittent and bolus + continuous-infusion PPIs are limited by ethno-geographical differences and differences in care processes and facilities.^9^ In addition, prior meta-analyses have been inconclusive due to methodologic issues of including patients without high risk, without endoscopic therapy or comparing high-low dose rather than continuous-intermittent infusion PPIs therapy.^10^ Thus, further studies are needed to confirm the possible ethno-geographical differences in patient outcomes using continuous versus (vs) intermittent PPIs infusion. In Saudi Arabia, the causes of upper gastrointestinal bleeding (UGIB) were predominantly nonvariceal with common incidences of rebleeding and mortality.^11,12^ This study aimed to compare the therapeutic effects of intermittent and continuous PPI infusions on early rebleeding, length of hospitalization, and mortality in patients with NVUGIB.

## Methods

A retrospective observational study was carried out using data obtained from the health records of patients admitted with NVUGIB who received continuous or intermittent PPI therapy from January 2013 until October 2019 at King Abdulaziz University Hospital (KAUH), Jeddah, Saudi Arabia. The continuous PPI infusion regimen was defined as the standard PPI bolus plus continuous infusion recommended by the current guidelines:^9^ an 80 mg intravenous (IV) bolus followed by a continuous 8 mg/h IV infusion over 72 hours. In comparison, intermittent PPI therapy was defined as PPIs administered in intermittent boluses. Because the degree of acid suppression required to reduce rebleeding is unknown, there were no constraints on the number of boluses given (such as once daily or more often), the doses (different doses per bolus), or the route of administration (oral vs intravenous). Therefore, patients who receive intermittent PPI therapy are given a dose of PPI at predetermined intervals and are not attached to infusion equipment (such as 80 mg bolus and 40-80 mg every 12 hours). Convenience sampling was used to recruit patients for the study. Studies on PPI use in NVUGIB were retrieved and extensively reviewed from online academic databases, including PubMed and Google Scholar, and gaps relating to intermittent or continuous PPI were identified. The strengthening the reporting of observational studies in epidemiology criteria was used to report the findings.

The study involved adult patients with a confirmed diagnosis of NVUGIB who received either intermittent or continuous PPI therapy. The outcomes measured were rebleeding rates within 30 days post-treatment (primary), the length of hospital stays and mortality rates (secondary). Fresh hematemesis or melena accompanied with the development of shock (pulse >100 beats/min, systolic blood pressure 100 mmHg) or a drop in haemoglobin concentration >2 g/dL for >24 hours were considered rebleeding.^13^ The length of hospital stays was defined as the date of hospital admission to discharge. Finally, all-cause in-hospital mortality is any death occurring during admission within 30 days.

The study included all adult patients aged 18 years and above with confirmed diagnoses of NVUGIB who received either intermittent or continuous PPI therapy at KAUH between January 2013 and October 2019. Patients with gastritis, duodenitis, esophagitis, lower GI bleeding or malignancy like cancer were excluded from the study.

The study’s minimum required sample size was estimated with the Epi Info™ application version 7.2.5.0 (Centre for Disease Control and Prevention, Atlanta, Georgia, USA).

Minimum sample size = [DEFFxNp(1-*p*)]/[(d^2^/Z^2^_1_ − _a/2_x(N-1)+*p*(1−*p*)]=85, where N is the population size (N=500), *p* is the hypothesized (%) outcomes frequency in the population (50%±5%), d is the 100% confidence limit (absolute +/%); 5%), the design effect (DEFF) is 1; and Z is a constant of 1.96, with a 95% confidence interval (CI).

### Statistical analysis

The study data were computed in Microsoft Excel (Windows 10, Microsoft) and transferred into the Statistical Package for the Social Sciences, version 28.0 (IBM Corp., Armonk, N.Y., USA) for statistical data analysis. A normality check was carried out using the Kolmogorov-Smirnov test and histogram on the continuous variables. Continuous variables were presented as mean (±SD) and categorical variables as frequencies (percentages). An Independent sample t-test was used to test the mean differences of continuous variables between the 2 treatment groups, while the Chi-square test was used to test the associations between the treatment groups and outcomes. A *p*-value of <0.05 was considered statistically significant and presented with the corresponding 95% confidence interval (CI).

Ethical approval was obtained from the institutional review board of KAU (Reference No. 773-19). The institutional or national research committee ethical standards, as well as the 1964 Declaration of Helsinki and its subsequent revisions or equivalent ethical standards were followed in this study involving human participants. Throughout the study, the study data’s identity, confidentiality, and privacy were assured and preserved.

## Results

Ninety-seven patients with NVUGIB who received intermittent (n=56) and continuous (n=41) PPI infusions therapy were included in the final analysis. The average (± SD) age (*p*=0.116) and gender distribution (*p*=0.345) were comparable. Hypertension 59 (60.8%) is the most common risk factor among the patients, followed by diabetes 47 (48.5%) and ischemic heart disease 22 (22.7%). Other important risk factors common among the patients were chronic kidney disease and cerebrovascular accidents. The endoscopic findings showed that most patients had stage III (lesion without active bleeding) NVUGIB (Table 1).

**Table 1:** - Socio-demographic and clinical characteristics of patients with nonvariceal upper gastrointestinal bleeding (N=97).

Variables	Type of PPIs Regimen		*P*-value
	Intermittent (n=56)	Continuous (n=41)	
Age (years; mean ± SD)	66.0 ± 16.1	58.0 ± 19.5	0.116
***Gender***			
Male	19 (33.9)	13 (31.7)	0.345
Female	37 (66.1)	28 (68.3)	
***Comorbidities***			
Hypertension	4 (73.2)	18 (43.9)	0.401
Diabetes mellitus	30 (53.0)	17 (41.4)	
Chronic kidney diseases	9 (16.0)	5 (12.1)	
Ischemic heart disease	15 (26.7)	7 (17.0)	
Cerebrovascular accident	7(12.5)	7 (17.0)	
Peptic ulcer disease	5 (8.9)	8 (19.5)	
Heart failure	5 (8.9)	3 (7.3)	
***Endoscopic findings (Forrest classification)***			
Ia (Spurting)	0	1	
Ib (Oozing)	5	10	
IIa (Non-bleeding vessel)	3	6	
IIb (Adherent clot)	2	2	
IIc (Flat pigmented spot)	3	4	
III (Clean based ulcer)	43	18	

Values are presented as number and percentage (%). Continuous variables tested using independent sample t-test. Categorical variables tested using Chi-square test. Statistical significance at *p*<0.05. PPIs: proton pump inhibitors, SD: standard deviation

### Proton pump inhibitors regimen and patient outcomes

The clinical outcomes of the patients with NVUGIB are summarized in Table 2. The intermittent and continuous PPI infusions therapies are comparable, and no significant difference in patients’ outcomes, namely, recurrent bleeding within 30 days (*p*=0.396), mortality within 30 days (*p*=0.308), and the length of hospital stays (*p*=0.067).

**Table 2:** - Association between proton pump inhibitors regimen and patients’ outcomes

Outcomes	Overall (N=97)	Type of PPIs regimen		*P*-value
		Intermittent (n=56)	Continuous (n=41)	
Rebleeding	6 (6.1)	5 (8.9)	1 (2.4)	0.396
Mortality	4 (4.1)	1 (1.7)	3 (7.3)	0.308
Length of stay (days)	9	4	5	0.067

Values are presented as number and percentage (%). Categorical variables tested using Chi-square test. Statistical significance at *p*<0.05. PPIs: proton pump inhibitors

## Discussion

The present study compared intermittent and PPI infusion therapies in patients with NVUGIB and found similar effects on patient outcomes. The 2 treatment groups did not show significant differences in rebleeding, length of hospitalization, or mortality rates. The most common cause of NVUGIB was peptic ulcer disease, which resulted in substantial morbidity and mortality.^14,15^ In a similar study involving patients with NVUGIB, a higher rebleeding rate was observed with continuous compared to intermittent PPI therapy (33.8 vs 23.0%; *p*=0.012). However, after adjusting for co-founders, no difference in rebleeding rates among the groups (adjusted OR, 1.50 [95% CI, 0.91-2.50]). There was also no change in the length of hospital or ICU stays, discharge disposition, or in-hospital mortality.^16^

Furthermore, in another study, Mahajan carried out a trial in patients with peptic ulcer disease and upper GI bleeding who randomly received pantoprazole (either a continuous or intermittent therapy). Among 118 patients, 7 (5.9%) had rebleeding. Three (5.1%) of the 59 patients who received continuous regimens and 4 (6.8%) of the 59 patients who received intermittent regimens had rebleeding. This result was not statistically significant.^17^ Furthermore, Ibrahim et al^18^ carried out a randomized double-blind study to compare the advantage of high and standard- dose omeprazole (IV omeprazole 40 mg bolus dose once daily followed by normal saline infusion vs IV bolus of 80 mg omeprazole followed by 8 mg/h infusion) as prophylaxis against upper GIT bleeding in high risk critically ill patients. It was found that patients on high dose omeprazole had higher gastric pH, lower incidence of critical significant GIT bleeding, higher ICU stay Hb, lower number of RBCs transfusion and shorter ICU stay. However, a study by Khan et al^19^ demonstrated that patients who received bolus intravenous PPI medication had worse results, including a higher need for other therapies. Although the most recent guidelines advocate intermittent PPI medication, there is no agreement on which PPI agent, dose, or frequency should be used.^16^

Our study also showed no significant difference in mortality rate between the intermittent and continuous PPI therapy groups (1 patient [1.7%] vs 3 patients [7.3%], *p*=0.308). Hsu et al^20^ carried out a trial to compare 2 dosages of pantoprazole infusion for peptic ulcer bleeding. After successful endoscopic therapy, patients with peptic ulcers and bleeding were enrolled. For 3 days, the patients were given a continuous pantoprazole infusion at either 192 mg/day or 160 mg/day. The clinical outcomes of the 2 groups were compared over the course of 14 days, with recurrent bleeding being the primary objective. They found no significant difference in mortality rate between the 2 groups (1 vs 0, *p*>0.1).^20^ Also, our study showed no difference between the 2 groups in length of hospital stays (intermittent infusion vs continuous infusion: 4 days vs 5 days, *p*=0.067). Similarly, in a meta-analysis by Neumann et al^21^ that evaluated the length of hospital stay, no significant difference was found between the 2 regimens (mean difference: 0.26 days; 95% CI, −0.08 to 0.6 days).

Intermittent therapy could serve as a cost-effective alternative to continuous intravenous PPI therapy. According to a recent study, the timing or amount of PPI does not affect the cost and is significantly less important than effective patient triage.^2^ A meta-analysis found that intermittent PPI use was non-inferior to bolus plus continuous infusion of PPIs in rebleeding, blood transfusion, hospitalization, and death.^5^ Also, intermittent oral PPIs may be equally successful in controlling pH as intermittent intravenous PPIs. This is contrary to managing chronic diseases like ischemic heart disease and mental disorders with prolonged treatment courses.^22,23^

### Study limitations

The present study has identified some limitations. First, this was a single-center retrospective cohort study with a small number of patients; thus, the study is not generalizable but would serve an essential role in building an expanded study. Second, the reason for individual treatment selection and respective PPI doses could not be established. Other factors that could influence patients’ outcomes, such as comorbidities and other medications that cause upper GI bleeding, like nonsteroidal anti-inflammatory drugs, antiplatelet, and antithrombotic agents, were not accounted for. Finally, most participants were Grade III with no sign of hemorrhage-clear base, indicating a selection bias. A national registry would allow for a better understanding of causes, risk factors, and other relevant patient-reported outcomes to provide better value-based treatment.

In conclusion, intermittent and continuous PPI infusions in patients with NVUGIB showed comparable effects on rebleeding, length of hospitalization, and mortality rates in patients attending the university hospital in Saudi Arabia. Further cost analysis of the 2 PPIs infusion methods could provide additional information for the health system and policy improvement.
